# The difficult laparoscopic cholecystectomy: a narrative review

**DOI:** 10.1186/s12893-025-02847-3

**Published:** 2025-04-12

**Authors:** Hamdy S. Abdallah, Mohamad H. Sedky, Zyad H. Sedky

**Affiliations:** 1 Faculty of Medicine, Tanta University, Tanta, Egypt; 2 Department of General Surgery, Tanta University Teaching Hospital, Al Geish St, Tanta, Gharbia 31527 Egypt; 3 Kasr-Alainy Faculty of Medicine, Cairo University, Cairo, Egypt; 4 Kasr-Alainy Faculty of Medicine, El Saray St, El Manial, Old Cairo, 11956 Egypt

**Keywords:** Safe laparoscopic cholecystectomy, Difficult laparoscopic cholecystectomy, Acute cholecystitis, Bile duct injuries, Intraoperative cholangiography, Bailout procedure, Subtotal cholecystectomy

## Abstract

**Background/purpose:**

Laparoscopic cholecystectomy is one of the most commonly performed general surgical procedures. Difficult laparoscopic cholecystectomy is associated with increased operative time, hospital stay, complication rates, open conversion, treatment costs, and mortality. This study aimed to provide a comprehensive literature review on difficult laparoscopic cholecystectomy.

**Methods:**

A literature search was conducted for articles published in English up to June 2024 using common databases including PubMed/MIDLINE, Web of Science, Google Scholar, and ScienceDirect. Keywords included “safe laparoscopic cholecystectomy”, “difficult laparoscopic cholecystectomy”, “acute cholecystitis”, “prevention of bile duct injuries”, “intraoperative cholangiography,” “bailout procedure,” and “subtotal cholecystectomy”. Only clinical trials, systematic reviews/meta-analyses, and review articles were included. Studies involving children, robotic cholecystectomy, single incision laparoscopic cholecystectomy, open cholecystectomy, and cholecystectomy for indications other than gallstone disease were excluded.

**Results/discussion:**

Emergency laparoscopic cholecystectomy for acute cholecystitis is ideally performed within 72 h of symptom onset, with a maximum window of 7–10 days. Intraoperative cholangiography can help clarify unclear biliary anatomy and detect bile duct injuries. In the “impossible gallbladder”, laparoscopic cholecystostomy or gallbladder aspiration may be considered. When dissection of Calot’s triangle is deemed hazardous or impossible, the fundus-first approach allows for completion of the procedure with either total cholecystectomy or subtotal cholecystectomy. Subtotal cholecystectomy is effective in preventing bile duct injuries, can be performed laparoscopically, and is currently the best available bailout approach for difficult laparoscopic cholecystectomy.

**Conclusion:**

Difficult laparoscopic cholecystectomy is a common clinical scenario that requires a judicious approach by experienced surgeons in appropriate settings. When difficult laparoscopic cholecystectomy is encountered, various bailout strategies are available. Currently, subtotal cholecystectomy is likely the most effective bailout approach.

## Highlights

Difficult laparoscopic cholecystectomy is associated with multiple negative surgical outcomes including a higher rate of bile duct injury.

Difficult laparoscopic cholecystectomy can be predicted before and at the start of surgery.

Several bailout procedures are available when difficult laparoscopic cholecystectomy is encountered.

Subtotal cholecystectomy is currently the best available bailout approach to difficult laparoscopic cholecystectomy.

## Introduction

Laparoscopic cholecystectomy (LC) is one of the most commonly performed general surgical procedures worldwide. In the United States, approximately 20 million people have gallstones, and between 300,000 and 750,000 cholecystectomies are performed annually [1, 2]. In Europe, approximately 900,000 cholecystectomies are conducted each year [3].

LC encompasses a wide spectrum of technical difficulties. At the easier end of the spectrum, it is a rapid and uncomplicated procedure, typically finished within an hour; however, at the more difficult end, it can present significant surgical challenges [4]. LC is also the leading cause of bile duct injury (BDI), which predominantly occurs when the procedure is complicated by inflammation or scaring.

Difficult LC is associated with increased operative time, blood loss, hospital length of stay, complication rates, conversion to open surgery, treatment costs, and mortality. Although difficult LC is reported in up to 26% of large series [5], there is no universally accepted definition of “difficulty” that would enable surgeons to predict its existence, implement strategies to mitigate it and anticipate outcomes and complications. According to Laws et al. [6], LC for a difficult gallbladder carries a higher surgical risk compared to standard LC. Difficult gallbladder is typically associated with severe inflammation that distorts local anatomy, making dissection more challenging and hazardous (e.g., acute calculous cholecystitis, empyema, gangrene, perforation, and Mirizzi syndrome) or with liver cirrhosis, which increases the risk of bleeding. Ashfaq et al. [7] defined a “bad gallbladder” as one that is necrotic, gangrenous, or perforated; has Mirizzi syndrome; exhibits extensive adhesions obscuring local anatomy; has a prior tube cholecystostomy; requires an operation lasting more than 120 min; or necessitates conversion to open surgery. The Tokyo Guidelines 2018 (TG18) stratified patients with acute cholecystitis into grades III, II, and I based on diagnostic criteria and a severity grading system [Table 1] [8]. By combining this grading system with the American Society of Anesthesiologists physical status classification and the Charlson comorbidity index, surgeons can determine the most appropriate management strategy for each patient based on disease severity [9].

**Table 1:** TG18/TG13 severity grading for acute cholecystitis [8]

**Grade III (Severe)**	Associated with dysfunction of any of the following organs/ systems: 1. Cardiovascular dysfunction (hypotension requiring treatment with dopamine 5 µg/kg per min or any dose of norepinephrine) 2. Neurological dysfunction (decreased level of consciousness) 3. Respiratory dysfunction (Pa02/Fi02 ratio 300) 4. Renal dysfunction (oliguria, creatinine 2.0 mg/dl) 5. Hepatic dysfunction (PT-INR 1.5) 6. Hematological dysfunction (platelet count 100,000/mm^3^)
**Grade II** **(Moderate)**	Associated with any one of the following conditions: 1. Elevated white blood cell count (18,000/mm^3^) 2. Palpable tender mass in the right upper abdominal quadrant 3. Duration of complaints 72 h 4. Marked local inflammation (gangrenous cholecystitis, pericholecystic abscess, hepatic abscess, biliary peritonitis, emphysematous cholecystitis)
**Grade I** **(Mild)**	Does not meet the criteria of grade II or grade III acute cholecystitis Can also be defined as acute cholecystitis in a healthy patient with no organ dysfunction and mild inflammatory changes in the GB

TG Tokyo guidelines, Pa02 partial pressure of Oxygen in arterial blood, Fi02 Fraction of inspired Oxygen, PT-INR Prothrombin time/International normalized ration, GB gallbladder

Since surgical outcomes depend on the interaction between the surgeon and the patient’s tissues, both factors play a critical role in the success of the procedure. An easy LC is the one that can be completed laparoscopically by a surgeon without specialist hepatobiliary training within a reasonable timeframe (< 90 min), without complications, the need for bailout procedures, or conversion to open surgery. In contrast, a difficult LC can be defined as a procedure that cannot be completed laparoscopically by surgeons without specialist hepatobiliary training without assistance, requires a longer operative time (> 90 min), results in surgical complications (including BDI), or necessitates a bailout procedure or conversion to open surgery.

The objective of this article is to provide an updated and comprehensive review of the literature relevant to difficult LC. This review covers essential topics such as relevant surgical anatomy, fundamental principles of safe LC, the mechanism of bile duct injuries (BDIs), preoperative and intraoperative prediction of difficulty, various approaches to managing difficult LC, and strategies for addressing BDI identified during surgery.

## Methods

**Fig. 1:**
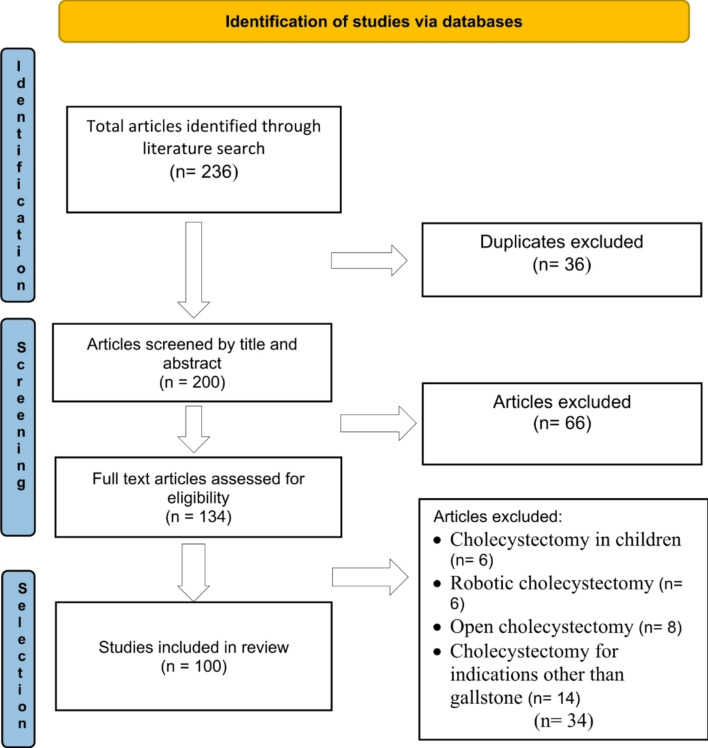
PRISMA study selection flow chart

A comprehensive literature search was conducted for articles published in English up to June 2024 using major databases, including PubMed/MEDLINE, Web of Science, Google Scholar, and ScienceDirect. The search employed the following keywords: “safe LC” “difficult LC”, “acute cholecystitis”, “prevention of BDIs”, “intraoperative cholangiography”, “bailout procedure” and “subtotal cholecystectomy (SC)”. Only clinical trials, systematic reviews, meta-analyses, and review articles were included. Studies involving children, robotic cholecystectomy, open cholecystectomy, and cholecystectomy for indications other than gallstone disease were excluded (Fig. 1).

## Results and discussion

The literature search identified 236 articles. After removing 36 duplicates, 200 articles remained. Initial screening of titles and abstracts led to the exclusion of 66 articles. A subsequent review of the remaining 134 full-text articles for eligibility resulted in the exclusion of an additional 34 articles. Ultimately, 100 articles were included in this review.

### Relevant surgical anatomy

The field of LC involves many critical structures, including major bile ducts and vascular structures within the portal triad, the porta hepatis, and the right portal pedicle, as well as adjacent organs such as the liver, duodenum, colon, and inferior vena cava. Therefore, it is imperative for the operating surgeon to have a thorough understanding of the relevant anatomy, common anatomical variations, and anatomical distortions caused by pathological processes such as acute and chronic cholecystitis.

#### The hepatocystic/calot’s triangle

The hepatocystic triangle is a triangular area on the undersurface of the liver, bounded medially by the common hepatic duct, caudally by the cystic duct, and cranially by the undersurface of segment 4. In his original description, Calot identified the cystic artery as the upper boundary of the “Calot’s triangle”. The hepatocystic triangle contains the cystic artery, a variable portion of the right hepatic artery, the cystic lymph node, and a variable amount of fibrofatty tissue [10]. During LC, this triangle must be dissected to achieve the critical view of safety (CVS) before dividing the cystic artery and cystic duct. The cystic lymph node, often located superficial to the cystic artery, serves as a key landmark for identifying the artery [11]. Inflammatory changes associated with acute and chronic cholecystitis can distort the triangle through adhesions, fibrosis, or scarring, complicating anatomical identification and dissection [12].

#### The cystic plate

The plate system is a connective tissue sheath that encloses the bile ducts and blood inflow vessels of the liver. The cystic plate, a component of this system, is a thin fibrous sheath located in the gallbladder bed and is continuous with the hilar plate and the Glissonian sheath of the right portal pedicle. During LC, as the gallbladder is dissected from its bed, the cystic plate should remain intact, appearing as a whitish or greyish structure in the gallbladder bed. It is essential to expose the lower part of the cystic plate as one of the three components of the CVS [13]. It is also crucial to stay close to the gallbladder wall during dissection to preserve the integrity of the cystic plate. Breaching the cystic plate can lead to several complications, including troublesome bleeding from the liver parenchyma, particularly if the terminal tributaries of the middle hepatic vein are injured. Also, a subvesical bile ducts that course superficially in the GB bed may be injured [14]. In some patients with chronic calcular cholecystitis, the gallbladder may become contracted and fibrotic, adhering densely to the cystic plate, which may also become short and thick. In the fundus-first (FF) approach, should the plane of dissection continue downward deep rather than superficial to the cystic plate, the fibrous sheath of the right portal pedicle will eventually be entered causing extreme vasculo-biliary injury [15].

#### Rouviere’s sulcus

First described by Henri Rouviere in 1924, this 2–3 cm sulcus is located on the undersurface of the right lobe of the liver, to the right of the liver hilum anterior to the caudate process. It typically contains the right portal triad or its branches [16]. Identifiable in 82% of individuals [17], Rouviere’s sulcus serves as an important fixed anatomical landmark that helps orient the surgeon during LC. When Hartmann’s pouch is grasped and retracted, the safe zone of dissection lies cephalad to a line extending from the roof of Rouviere’s sulcus to the umbilical fissure across the base of segment 4. This safety line, known as the R4U line (Rouviere’s sulcus, Segment 4, Umbilical fissure), demarcates the safe zone (cephalad to the line) from the danger zone (caudad to the line) [12].

#### The cystic artery and right hepatic artery

In 75% of individuals, the cystic artery arises from the right hepatic artery, traverses Calot’s triangle, and divides into a superficial branch that runs along the peritoneal surface of the gallbladder and a deep branch that courses between the gallbladder and the cystic plate. If the dissection is close to the gallbladder and the branching is proximal, a posterior cystic artery may require separate ligation. The right hepatic artery typically courses behind the common hepatic duct before entering Calot’s triangle. A tortuous right hepatic artery is not uncommon, often forming a “Caterpillar turn” or “Moynihan’s hump” near the gallbladder before giving off a short cystic artery. This anatomical variation makes the right hepatic artery particularly susceptible to injury during cholecystectomy, and the surgeon should be vigilant for this possibility if an unusually “large cystic artery” is encountered [10].

### Safe laparoscopic cholecystectomy

BDIs are the most serious complications of LC. They are often very morbid and may lead to death. These injuries often necessitate additional surgical, radiologic, or endoscopic interventions. Moreover, they are frequently associated with extended hospital stays, prolonged convalescence, loss of work time, and impairment of the patient’s quality of life [18]. Additionally, BDIs frequently result in litigation. Therefore, preventing BDIs is a primary objective of LC [2].

In 2019, Strasberg proposed a three-step conceptual roadmap to prevent BDI during LC. These steps include: (1) getting a secure anatomical identification whenever conditions permit; (2) recognizing when conditions are too hazardous to get secure anatomical identification (inflection point); and (3) finishing the procedure safely when secure anatomical identification is not possible (bailout procedures) [4].

#### The critical view of safety (getting secure anatomical identification)

The primary mechanism underlying most major BDIs is the misidentification (or misperception) of anatomical structures at or near the hepatocystic triangle [19, 20]. Specifically, the common bile duct or an aberrant right sectional duct may be mistakenly identified as the cystic duct and subsequently clipped and divided. Similarly, the right hepatic artery may be misidentified as the cystic artery if the right hepatic artery follows an aberrant course and the cystic artery is short. To prevent such misidentification injuries, it is critical to conclusively identify the cystic duct and cystic artery before clipping and dividing them [2, 12].

The CVS is a standardized method for the secure identification of cystic structures. To achieve the CVS, three requirements must be completed [13]. First, the lower third of the GB must be dissected free from the cystic plate. Second, the hepatocystic triangle must be cleared of all fibrofatty tissue. Third, finding two and only two structures - the cystic duct and cystic artery - entering into the gallbladder. The CVS must be seen clearly both from front and back to have complete circumferential visualization of cystic duct and cystic artery (doublet view) [2]. Once the CVS is achieved, the surgeon should pause (time-out 4) to reconfirm its attainment. At this stage, the CVS can be documented by photographs and/or video recordings for verification [21].

Evidence strongly supports that achieving the CVS is an effective method of accurately identifying anatomical structure at the Calot’s triangle significantly reducing the incidence of BDIs [22]. Notably, in cases of major biliary injuries, the CVS is rarely achieved [23]. Therefore, difficulty in achieving the CVS should be regarded as a critical warning sign, indicating that further dissection may be hazardous posing an increased risk of biliary and/or vascular injury (reaching the inflection point) [2, 4, 24]. In such scenarios, alternative strategies (bailout techniques) should be considered to safely complete the procedure [2, 4].

#### The concept of time out

During difficult LC procedures, surgeons may become disoriented, potentially entering the “zone of danger.” To mitigate this risk, the concept of “time-out” has been introduced. During a time-out, the surgeon should pause, reorient himself, take stock of the situation, and then proceed [12]. To reorient, the surgeon should identify five fixed anatomical landmarks, collectively referred to as “B-SAFE” by Sutherland and Ball [25]: B (bile duct), S (Rouviere’s sulcus and segment 4), A (hepatic artery), F (umbilical fissure), and E (enteric structures, including the duodenum, colon, and stomach). To do so, the cameraman should withdraw the laparoscope to provide a panoramic view of the surgical field. Time-outs should be routinely performed at the following stages of the procedure: (1) immediately after entering the abdomen; (2) before dissecting the hepatocystic triangle; (3) when encountering anatomical ambiguity, anomalies, or any uncertainty or difficulty; and (4) before clipping and dividing the cystic duct and cystic artery after achieving the CVS [12, 25].

### The problem of bile duct injury

#### Incidence of bile duct injury

Given the vast number of LCs performed worldwide each year, thousands of patients experience BDIs, with their subsequent severe and long-term health implications. BDIs also significantly impact surgeons’ mental well-being and professional reputation and impose a substantial financial burden on healthcare systems [26]. According to large nationwide databases and systematic reviews, major BDIs occur in 0.1% of elective LCs and 0.3% of emergency LCs. When considering all types of BDIs, the rates rise to 0.4% for elective LCs and 0.8% for emergency LCs [27]. Conner and Garden [28] quoted that nearly one-third to one-half of surgeons in the United States and British Columbia have caused a major BDI, with an individual experience of 1–2 such cases. Khan et al. [29] investigated the rate of BDIs referred for endoscopic retrograde cholangiopancreatography (ERCP). Among 17,684 ERCP records, 183 patients were identified with BDIs. The study concluded that the frequency, anatomic distribution, and rate per 100 ERCPs remained consistent over time. Sedlack [30] analyzed the issue of BDI from a quality improvement perspective. He postulates that if one major BDI per 1500 LC is accepted, and Six Sigma principles are applied, this would equate to 95 defects per million opportunities (DPMO - a metric that tells how good a process is towards committing mistakes) and a Sigma level of 5.25. If this defect rate is applied to the airline industry, this would equate to 20 commercial airline crashes per day in the United States alone!

#### Pathogenesis of bile duct injury

Certainly, coexisting acute or chronic inflammation around the gallbladder and hepatoduodenal ligament, poor exposure and bleeding in the surgical field, and anatomical variations of the bile ducts and hepatic arteries increase the difficulty of the surgical procedure and significantly increase the risk of BDI. Studies have proved, however, that misinterpretation of biliary anatomy is the primary cause of BDI in 71–97% of cases [31].

#### Prevention of bile duct injury

Approximately 70% of surgeons consider BDI unavoidable [32]. While most injuries occur during the surgeon’s learning curve, about one-third of injuries occur after more than 200 LCs, suggesting that factors beyond surgical experience contribute to BDI [33]. Among these factors, misidentification of biliary anatomy is the most important.

Several techniques were proposed to prevent BDIs, with the most critical being the establishment of the CVS. Additional preventive measures include the use of a 30° telescope, avoiding the use of diathermy near the bile ducts, and minimizing unnecessary dissection near the cystic duct-common hepatic duct junction [34]. During surgery for acute cholecystitis, suctioning the tense and distended gallbladder enhances the ability to grasp and retract it effectively. Dissection using the tip of the suction/irrigation cannula is particularly useful, as it enables safe blunt dissection along anatomical planes while simultaneously clearing blood and fluid from the surgical field, enhancing visualization of the critical anatomical structures. In case of bleeding, blind clipping or application of energy should be avoided. Instead, gauze compression directly over the bleeding point, combined with suctioning the collected blood, should be applied for 1–2 min. This technique will control oozing surfaces, allowing clear identification of any bleeding vessels or non-bleeding stumps, which can then be selectively controlled.

The Society of American Gastrointestinal and Endoscopic Surgeons (SAGES) has proposed strategies to foster a universal culture of safety and minimize the risk of BDI during LC. These strategies include: (1) utilizing the CVS for identification of cystic duct and cystic artery, (2) acknowledging the potential for aberrant anatomy in all cases, (3) employing liberal use of cholangiography or other methods to image the biliary tree intraoperatively, (4) considering an intraoperative momentary pause before clipping, cutting or transecting any ductal structures, (5) recognizing when the dissection is approaching a zone of significant risk and halt the dissection before entering the zone, (6) finishing the procedure by a safe alternative if conditions around the gallbladder are too hazardous and (7) seeking help from another surgeon when facing difficult dissection or a challenging situation [35]. The World Society of Emergency Surgery (WSES) 2020 guidelines align with these recommendations [27].

### Causes (predictors) of difficult laparoscopic cholecystectomy

A scoring system to preoperatively predict the difficulty level of LC would enhance patient selection for day-case surgery, optimize preoperative planning, and improve patient counseling regarding the risks of the procedure. Such a system would also help determine whether specialized equipments (e.g., fluorescence cholangiography) or the presence of an expert surgeon in the operating room is necessary. Additionally, it would be valuable for designing resident training programs [36, 37].

Over the years, several scoring systems have been developed to predict the difficulty of LC. These systems evaluate several clinical, radiological, biochemical, and operative predictors of difficulty [12] [Table 2]. Radiologists have attempted to establish preoperative ultrasound (US) scores to assess the risk of intraoperative difficulty during LC. Siddiqui et al. (2017) [38] developed an US-based scoring system that includes gallbladder wall thickness, transverse diameter, pericholecystic collection, number and mobility of gallbladder stones, common bile duct diameter, and liver size. A score > 5 demonstrated 80.7% sensitivity and 91.7% specificity for identifying difficult LC.

**Table 2:** Predictors of difficult gallbladder [12]

**History** Male Age > 65 year) Prior AC, Interval between onset and presentation > 72–96 h in AC Previous multiple attacks of biliary pain (> 10) History of AC Upper abdominal surgery Prior attempt at cholecystectomy (including cholecystostomy)	
Physical examination Fever Higher ASA score Morbid obesity	
Laboratory tests Raised WBC count (> 18000/mm3) Raised CRP	
**Imaging (USG/CT/MRCP)** Thick walled GB (> 4–5 mm) Small contracted GB Distended GB with a stone impacted in neck Gangrenous/perforated GB Mirizzi syndrome/Cholecystoenteric fistula Cirrhosis/portal cavernoma with portal hypertension	
**Intraoperative** Small shrunken GB not visualized on initial exploration Liver edge puckering near fundus Fatty or cirrhotic liver (difficulty in retraction)	

AC acute cholecystitis, ASA American Society of Anaesthesiology, WBC white blood cell, CRP C-reactive protein, US Ultrasonography, CT computerized tomography, MRCP magnetic resonance cholangio-pancreatography, GB gallbladder

In 2009, Randhawa and Pujahari [39] introduced a 15-point scale for the preoperative prediction of difficult LC. This model incorporated variables such as age, sex, history of hospitalization for acute cholecystitis, body mass index, abdominal scarring, palpable gallbladder, thick-walled gallbladder, and impacted stones. Difficult LC was defined based on intraoperative endpoints, including operative time, bile/stone spillage, BDI, and conversion to open surgery. The Randhawa score accurately predicted easy and difficult cases in 88.8% and 92% of cases, respectively. Independent predictors of difficult LC included body mass index > 27.5, previous hospitalization for acute cholecystitis, palpable gallbladder, and thick-walled gallbladder in the US. In 2013, Gupta et al. [40] validated the Randhawa scoring system, identifying four key predictors of difficult LC: history of hospitalization, palpable gallbladder, impacted stones, and gallbladder wall thickness in the US. The score achieved 95.7% sensitivity and 73.7% specificity, with positive predictive values of 90% and 88% for easy and difficult cases, respectively. The area under the receiver operating characteristic (ROC) curve was 0.86. Two additional studies further confirmed the validity of the Randhawa score for predicting difficult LC [41, 42].

In 2017, Iwashita et al. [43] conducted a Delphi consensus process among expert surgeons from Taiwan, Japan, and Korea to evaluate 25 intraoperative findings associated with difficult LC. Each factor was graded on a seven-point scale (0–6). Cholecystoenteric fistulas were rated as the most challenging (median score, 6), followed by diffuse scarring in Calot’s triangle (median score, 5), necrotic changes (median score, 4), abscess formation (median score, 4), gallbladder inversion (median score, 4), collateral vein formation due to liver cirrhosis (median score, 4), and anomalous bile ducts (median score, 4).

A recent Delphi study aimed to establish a consensus among Spanish surgical experts on the criteria defining difficult LC. The criteria that reached consensus included BDI (96.77%), non-evident anatomy (93.55%), Mirizzi syndrome (93.55%), severe inflammation of Calot’s triangle (90.32%), conversion to laparotomy (87.10%), time since the last episode of acute cholecystitis (83.87%), scleroatrophic gallbladder (80.65%), and pericholecystic abscess (80.65%) [36].

In 2020, Nassar et al. [37] developed a preoperative scoring system to predict difficult LC defined by an intraoperative difficulty grading scale that underwent internal and external validation. The authors identified several independent predictors of difficulty. The score achieved an area under the ROC curve of 0.789 on external validation. Patients were stratified into low (scores 0–1), medium (scores 2–6), and high (scores 7+) risk categories, with 11.0% of low-risk and 80.0% of high-risk patients experiencing difficult surgeries [Table 3].

**Table 3:** Nassar risk score [37]

Age (years)	
< 40	0
40+	1
Gender	
Female Male	0 1
ASA Classification	
1 2 3 4–5	0 1 2 7
Primary diagnosis	
Pancreatitis Biliary Colic CBD stone Cholecystitis	0 0 1 4
Thick-walled GB (≥ 3 mm)	
No Yes	0 2
CBD dilation (> 6 mm)	
No Yes	0 1
Pre-operative ERCP	
No Yes	0 1
Admission type	
Elective Delayed Emergency	0 1 2

ASA American Society of Anaesthesiology, CBD common bile duct, ERCP endoscopic retrograde cholangio-pancreatography

The Tokyo Guidelines (TG13) diagnostic criteria and severity grading for acute cholecystitis have been extensively validated and are significantly associated with 30-day overall mortality, hospital length of stay, conversion to open surgery, and treatment costs. Consequently, the TG13 criteria were adopted unchanged as TG18/TG13 [Table 1] [8].

In summary, the most frequently reported preoperative predictors of difficult LC include advanced age, obesity, history of previous upper abdominal surgery, recurrent episodes of cholecystitis, leukocytosis, thick-walled gallbladder, pericholecystic fluid collection, distended or contracted gallbladder, stones impacted at the gallbladder neck, and Mirizzi syndrome.

### Intraoperative predictors of difficult laparoscopic cholecystectomy

As previously mentioned, surgeons should have an initial time-out upon entering the abdomen to assess anatomical landmarks and identify signs of potential technical difficulty. These predictors include omental or bowel adhesions (e.g., duodenum/transverse colon), distended, edematous, or thick-walled gallbladder, scarred or fibrotic gallbladder, the Pucker sign, cirrhotic liver with laterally displaced gallbladder, pericholecystic abscess, obliterated or scarred hepatocystic triangle due to biliary inflammatory fusion, intrahepatic gallbladder, obscured anatomical landmarks, dilated veins in the hepatoduodenal ligament, and others.

### The asymptomatic gallbladder

Although DLC is typically associated with acute and chronic cholecystitis, intraoperative challenges may also arise during LC for asymptomatic gallbladder stones. These challenges can stem from factors such as anatomical variations, obesity, prior abdominal surgery, intra-abdominal inflammation, or liver cirrhosis. In such cases, the adage that “the best way to avoid complications of a certain procedure is NOT to perform that procedure” holds particular relevance. A medical intervention is deemed appropriate only when its benefits significantly outweigh its risks, making the procedure worthwhile. Thus, establishing a correct indication is a fundamental principle in surgery. Specifically, the necessity of LC must be critically evaluated on a case-by-case basis, particularly when the procedure is anticipated to be difficult or hazardous [44].

The low likelihood of symptoms (< 20%) or complications in patients with asymptomatic gallbladder stones is often outweighed by the risks associated with surgery and the additional healthcare costs. Consequently, current practice guidelines — primarily based on the natural history of asymptomatic gallstones rather than randomized clinical trials comparing non-operative management with LC — recommend against routine LC for asymptomatic cholelithiasis [45]. With few exceptions, the risks of surgery for asymptomatic stones generally exceed the risks of complications or development of gallbladder cancer. Exceptions include patients with sickle cell disease, those on long-term total parenteral nutrition, individuals who are therapeutically immunosuppressed following solid organ transplantation, and those without immediate access to healthcare facilities. Additionally, LC may be justified for gallbladder polyps measuring 1–10 mm in diameter, porcelain gallbladder, and certain ethnic groups with a high risk of gallbladder cancer [46, 47]. Incidental LC is also considered acceptable during other laparoscopic procedures, such as Roux-en-Y gastric bypass.

### Different approaches to difficult laparoscopic cholecystectomy

**Fig. 2:**
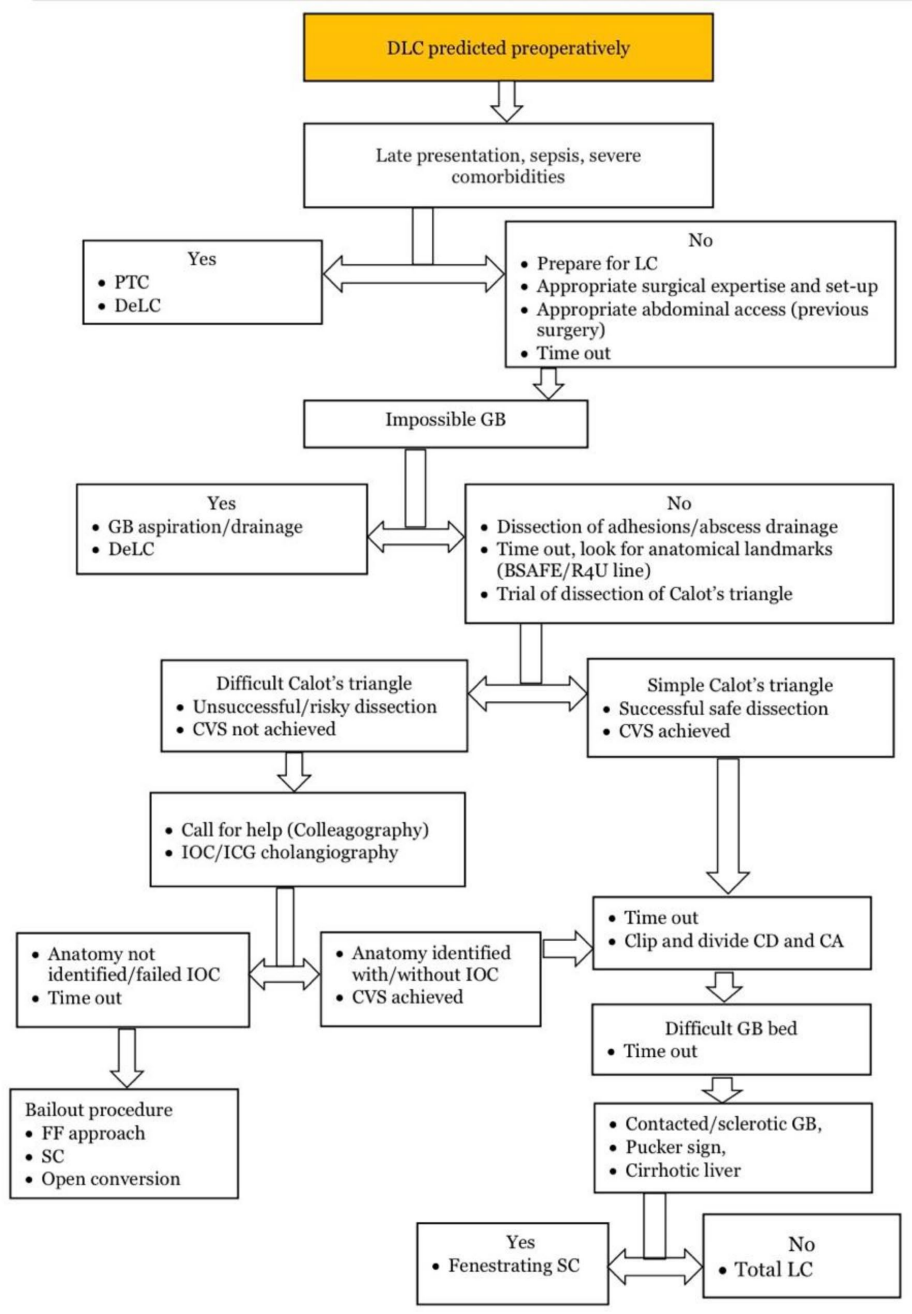
A flow-chart for the approach to a DLC DLC: difficult laparoscopic cholecystectomy, DeLC: delayed laparoscopic cholecystectomy, PTC: percutaneous transhepatic cholecystostomy, LC: laparoscopic cholecystectomy, GB: gallbladder, BSAFE B: bile duct, base of segment 4, S: Rouviere’s sulcus, segment 4, A: hepatic artery, F: umbilical fissure, E: enteric structures, R4U: Rouviere’s sulcus, 4 base of segment 4, U umbilical fissure, CVS: critical view of safety, IOC: intraoperative cholangiogram, ICG: Indocyanine green, CD: cystic duct, CA: cystic artery, SC: subtotal cholecystectomy

As a general principle, difficult LC should not be performed by surgeons in training or those with limited surgical experience. When difficult LC is anticipated based on preoperative data/scores or intraoperative findings, only surgeons with advanced laparoscopic skills and substantial expertise in biliary surgery should undertake these procedures. A flowchart outlining the approach to difficult LC is provided (Fig. 2).

#### Avoid surgery for late-presenting acute cholecystitis

A substantial body of evidence demonstrates that immediate or early LC yields superior or at least non-inferior outcomes compared to delayed LC in patients with acute cholecystitis, concerning morbidity, mortality, conversion rates, length of stay, and hospital costs. Cao et al. (2016) [48] published a meta-analysis of 77 case-control studies involving 40,910 patients. This study found that patients in the urgent or early LC group, compared to those in the delayed LC group, had a significantly lower incidence of BDI, bile leakage, conversion to open surgery, wound infection, blood loss, and length of stay. The lower incidence of BDI in early LC for acute cholecystitis was further corroborated by an analysis of the Swedish GallRiks Registry, which showed that the incidence of BDI increased progressively with the time interval from disease onset to cholecystectomy: 0.17% on the day of admission, 0.67% three days after admission, and 0.93% five days or later. Additionally, the 30-day and 90-day mortality rates significantly increased when surgery was performed after the fourth day of admission [49]. A more recent meta-analysis by Borzellino et al. (2021) [50] found that immediate LC (performed within 24 h of admission) did not reduce postoperative complications unless performed within 72 h of symptom onset.

The updated 2020 guidelines of the WSES recommend that, in the presence of adequate surgical expertise, early LC should be performed within seven days of hospital admission and ten days of symptom onset [51]. The Tokyo Guidelines 2018 (TG18) provided a flowchart for managing patients with acute cholecystitis based on disease severity. For Grade I patients, TG18 recommends performing LC within 72 h if possible or within one week if the patient is fit for surgery. For Grade II patients, LC should be performed in an advanced surgical center soon after symptom onset, provided the patient is fit for surgery. Special care should be taken to avoid BDI, and conversion to open cholecystectomy or SC should be considered when necessary. For Grade III patients, efforts should be made to stabilize organ function if required. If the patient is fit for surgery, early LC can be performed by a specialist surgeon with extensive experience in a setting equipped for intensive care management. In Grades II and III, if the patient is unfit for surgery, conservative treatment, including early biliary drainage, should be pursued [8].

#### When a difficult laparoscopic cholecystectomy is predicted, open or laparoscopic approach?

Once the decision to proceed with surgery has been established, the next consideration should focus on the surgical approach: open or laparoscopic? The existing literature provides evidence-based guidance to answer this question. Teixeira et al. [52] analyzed the outcomes of 520 patients who underwent surgery for acute cholecystitis between 2007 and 2013: 412 underwent LC, and 108 underwent open cholecystectomy. The outcomes were as follows: mortality (0.7% vs. 3.7%, *p* = 0.0369), perioperative complications (3.6% vs. 12.9%, *p* = 0.0006), postoperative surgical complications (7.7% vs. 17.5%, *p* = 0.0055), main BDIs (0.9% vs. 1.8%, *p* = 0.6091), reoperation rates (2.9% vs. 5.5%, *p* = 0.2315), and length of stay up to four days after surgery (64.8% vs. 18.5%, *p* < 0.001). The conversion rate was 10.7% when surgery was performed more than four days after diagnosis (13.7% vs. 8.8%), and postoperative surgical complications were more frequent in the converted group (20.4% vs. 6.2%, *p* = 0.0034).

Coccolini et al. [53] published a systematic review/meta-analysis of 10 trials comparing open cholecystectomy and LC in patients with AC, involving 1,248 patients (677 in the LC group and 697 in the open cholecystectomy group). LC was associated with half the postoperative morbidity rate (OR = 0.46), lower rates of wound infection and pneumonia (OR = 0.54 and 0.51, respectively), a lower postoperative mortality rate (OR = 0.2), and a significantly shorter mean postoperative length of stay (MD = -4.74 days). There were no significant differences in bile leakage rates, intraoperative blood loss, or operative time. The authors concluded that cholecystectomy for acute cholecystitis should initially be attempted laparoscopically.

As previously mentioned, the TG18 [8] and the updated 2020 WSES guidelines [51] recommend starting with LC in patients with acute cholecystitis. Only critically ill patients, such as those presenting with septic shock or absolute contraindications to laparoscopy, should avoid LC.

#### Percutaneous transhepatic cholecystostomy

In patients with a high perioperative risk due to sepsis, late presentation, or underlying comorbidities, the initial treatment for acute cholecystitis is percutaneous transhepatic cholecystostomy tube placement, followed by delayed cholecystectomy performed at least 6–8 weeks later. Percutaneous transhepatic cholecystostomy, performed under ultrasound or computed tomography guidance, decompresses the gallbladder, alleviates pain, and may prevent complications such as Mirizzi syndrome, gangrene, or perforation resulting from ischemic changes in the gallbladder wall [54]. Approximately 80% of patients who undergo percutaneous transhepatic cholecystostomy for acute cholecystitis experience immediate clinical improvement [55]. For those who recover, the tube can be removed once it returns clear bile, cholecystography confirms a patent cystic duct, or when the tract matures, usually within four weeks. Elective LC can then be scheduled within 6–8 weeks, provided the patient has regained medical fitness [56]. LC can be successfully completed in most patients, with a conversion rate of 14–32% [57, 58], which is higher than the rates for elective (5%) [59] and emergent LC (6%) for acute cholecystitis [60]. Failure to improve after percutaneous transhepatic cholecystostomy may indicate gallbladder gangrene or perforation, mandating damage control surgery.

#### Call for help/second opinion

The operating surgeon should pause and seek a second opinion from another surgeon when encountering a difficult gallbladder. Since misidentification is the primary cause of biliary and vascular injuries, the involvement of a second surgeon can prevent such injuries in up to 18% of cases [61]. Additionally, a colleague can provide an objective external perspective, scrub to assist in the procedure, offer suggestions to overcome technical challenges, and support the decision to convert to an exit strategy [62]. This practice should be encouraged at all levels of surgical expertise and viewed as a hallmark of good surgical practice rather than a sign of incompetence [12].

### The role of intraoperative imaging

#### Intraoperative cholangiogram

The primary role of intraoperative cholangiogram in difficult LC is to delineate biliary anatomy and to prevent or identify BDIs. Despite extensive literature on this topic, the evidence remains conflicting. Critics argue that intraoperative cholangiogram is not cost-effective, prolongs operative time, and is not consistently effective in preventing or identifying BDIs [63]. In a recent study, Esposito et al. [64] reported that intraoperative cholangiogram was incomplete or unclear in 11% of patients and could not be performed in 15% of them. Furthermore, while three patients sustained BDIs, intraoperative cholangiogram failed to identify any of them. Sheffield et al. retrospectively analyzed data from 37,533 patients (280 with BDIs) who underwent LC with intraoperative cholangiogram for gallstones between 2000 and 2009. After controlling for confounders, no statistically significant association was found between intraoperative cholangiogram and BDI. The authors concluded that intraoperative cholangiogram is not an effective preventive strategy against BDI during LC [65]. A meta-analysis of 14 studies involving 440,659 patients, recently published by Hall et al. [66], found no statistically significant difference in the incidence of BDI between selective and routine intraoperative cholangiogram. Similarly, no statistically significant difference was observed in BDI detection rates between LC with and without intraoperative cholangiogram.

Proponents of intraoperative cholangiogram, however, highlight its potential to reduce the incidence of BDI, facilitate earlier recognition of injuries, and improve the success of subsequent repairs. Törnqvist et al. [67] analyzed data from 50,000 LCs (17.5% performed for acute cholecystitis) in the GallRiks registry from 2005 to 2010 to assess the role of intraoperative cholangiogram in preventing iatrogenic BDI. They found no preventive effect of intraoperative cholangiogram in uncomplicated gallstone disease, but it reduced the risk of BDI in patients with concurrent acute cholecystitis (OR 0.44, 95% CI 0.30–0.63) or a history of acute cholecystitis (OR 0.59, 95% CI 0.35–1.00). A recent systematic review/meta-analysis by Rystedt et al. (2023) [68] examined the rate and odds of BDI with selective or routine intraoperative cholangiogram during LC from 1990 to 2018. The analysis included over 2 million patients and 9,000 BDIs. The rate of BDI was 0.36% with routine intraoperative cholangiogram, compared to 0.53% with selective intraoperative cholangiogram, indicating a 43% increased risk of BDI when intraoperative cholangiogram was used selectively (odds ratio 1.43, 95% CI 1.22–1.67).

Despite its debated efficacy in preventing BDI, SAGES recommends that surgeons should use intraoperative cholangiogram liberally, be familiar with its indications, and become proficient in the technique and interpretation of cholangiographic images [69].

In this respect, it is important to emphasize that the timing of BDI recognition is a critical determinant of the outcomes of surgical repair. Therefore, the ability of intraoperative cholangiogram to detect BDIs during LC is likely its greatest benefit. Data from the GallRiks prospective registry revealed that patients with BDIs and delayed detection had double the mortality risk compared to those without BDIs, while the 1-year survival rate was similar between patients with perioperatively detected BDIs and those without BDIs [67].

Given the conflicting evidence, it is more logical and practical to use intraoperative cholangiogram selectively rather than routinely. In 2020, five international surgical societies—the SAGES, the American Hepato-Pancreato-Biliary Association, the International Hepato-Pancreato-Biliary Association, the Society for Surgery of the Alimentary Tract, and the European Association for Endoscopic Surgery —published multi-society practice guidelines on safe LC and the prevention of BDI. These guidelines strongly recommend the selective use of intraoperative cholangiogram in patients with uncertain biliary anatomy and in those with suspected BDI [70].

As highlighted earlier, intraoperative cholangiography is primarily employed during difficult LC to prevent or identify BDIs. However, it also serves as a valuable tool for detecting common bile duct stones, particularly in patients at a higher risk of choledocholithiasis. High-risk populations include those with a history of obstructive jaundice or cholangitis, patients recovering from pancreatitis, and patients with elevated serum bilirubin, alkaline phosphatase, and pancreatic enzymes levels. Additionally, patients exhibiting a dilated common bile duct on preoperative US or those undergoing LC following endoscopic management of common bile duct stones are also considered at increased risk. Notably, these are the same patients who are more likely to have a difficult LC. Donnellan et al. performed a meta-analysis of 62 studies investigating the use of intraoperative cholangiography during LC. Among these, 8 studies involving 4,556 patients reported the incidence of common bile duct stones detected via selective intraoperative cholangiography, with a mean detection rate of 3.9% (0.7-12.8%) [71].

#### Intraoperative fluorescence cholangiogram

Several studies suggest that the use of indocyanine green (ICG) near-infrared fluorescence cholangiography during LC is superior to white light alone in identifying extrahepatic biliary anatomy, thereby reducing the risk of BDI. However, the tissue penetration of near-infrared fluorescence is limited to 5–10 mm, which restricts its ability to delineate deeply located bile ducts, such as the common hepatic duct, or bile ducts embedded in thick connective tissue in patients with severe cholecystitis or obesity before dissection of Calot’s triangle [72–73]. Yoshiya et al. [74] retrospectively evaluated the use of ICG in a cohort of patients with acute cholecystitis following percutaneous transhepatic cholecystostomy. They found that the ICG group had a significantly shorter operative time (129 ± 46 vs. 150 ± 56 min, *p* = 0.0455), a markedly lower conversion rate (2.6% vs. 22.0%, *p* = 0.0017), and a lower proportion of SC (0% vs. 6.6%, *p* = 0.0359) compared to the non-ICG group. Serban et al. (2022) [75] published a systematic review of 19 articles involving 2,490 patients on the use of ICG in LC and/or robotic cholecystectomy. Overall, the conversion rate was 0.52% in the ICG group and 2.52% in the non-ICG group, with higher rates observed in patients with acute and complicated cholecystitis. Additionally, the incidence of BDI was 0.12% in the ICG group and 1.31% in the non-ICG group. Despite its potential benefits, the current level of evidence supporting the role of near-infrared fluorescence cholangiography using ICG in difficult LC remains limited, as well-designed prospective trials are still lacking. Therefore, near-infrared fluorescence cholangiography remains an investigational technique that may prove beneficial in the future.

### Bailout (damage control) procedures

#### Identifying the need for a bailout strategy

In the three-step roadmap proposed by Strasberg for safe LC, the second concept involves recognizing when conditions are too hazardous to achieve secure anatomical identification and deciding not to perform a total cholecystectomy laparoscopically. Strasberg termed this the “inflection point,” defined as the moment when the decision is made to abandon the attempt to perform a total cholecystectomy laparoscopically and to finish the operation using an alternative method. This point is typically reached due to acute or chronic inflammation, anatomical variations, previous upper abdominal surgery, or liver cirrhosis [4].

#### Abortion of cholecystectomy in the impossible gallbladder

If during LC, dissection is deemed impossible raising concerns of causing more harm than good, the safest bailout strategy is to abort the procedure entirely. A typical scenario is when only a portion of the gallbladder, usually the fundus, can be exposed after attempted dissection due to dense adhesions between the gallbladder and bowel or the presence of a cholecystoenteric fistula. This approach is also the fastest way to conclude the procedure in cases of severe acute cholecystitis and intraoperative hemodynamic instability. In such situations, one of two options may be considered. The first is laparoscopic cholecystostomy, which- like percutaneous cholecystostomy - serves as a temporizing measure to drain the gallbladder, control symptoms, and halt the active inflammatory process. Delayed cholecystectomy is typically performed at least six weeks later [76–77]. The second option is gallbladder aspiration without drainage. In 2020, Kharytaniuk et al. [78] published a retrospective analysis of all LCs attempted under one surgeon’s care over 19 years, comparing gallbladder aspiration as a bailout procedure with standard conversion to open cholecystectomy. Among 757 attempted LCs, 40 (5.3%) were deemed impossible gallbladders and underwent aspiration with antibiotic injection. Interval LC was successful in 29/40 (72.5%) patients. Overall, only 5/757 patients (0.66%) required open conversion, compared to 55/1209 (4.55%) LCs performed during the same period. No aspiration-related complications were reported and postoperative length of stay and costs were significantly lower in the aspiration group than in the converted group. The authors concluded that laparoscopic gallbladder aspiration is a safe bailout procedure for impossible gallbladders, particularly in critically ill patients and for surgeons with limited experience in open cholecystectomy.

The primary drawback of these bailout procedures is that patients will require another hospitalization for cholecystectomy, incurring additional costs. However, when there is a concern about BDI, any approach that prevents this catastrophic outcome should be considered acceptable.

#### Fundus-first approach (dome-down, top-down, retrograde approach)

When encountering a difficult LC due to severe inflammation or biliary inflammatory adhesions and scarring in Calot’s triangle, the fundus-first (FF) approach to LC may allow completion of the procedure while avoiding BDI, serving as an alternative to immediate conversion to open cholecystectomy. In 2022, two systematic review/meta-analyses by El-Boghdady et al. [79] and Garzali et al. [80] examined the utility of the FF approach in difficult LC. Both studies found that the FF approach, compared to the conventional anterograde approach, was associated with shorter operative times, a lower incidence of BDI, and reduced rates of conversion to open surgery. The authors concluded that the FF approach is a feasible and a safer alternative to conventional LC for difficult gallbladders.

In this context, it is important to note that the FF approach carries a potential risk (error trap) for the unwary surgeon. The cystic plate is anatomically attached to the anterior wall of the right portal pedicle. In patients with acute or chronic cholecystitis, the normal plane of cleavage between the gallbladder wall and the cystic plate may be obliterated due to adhesions and scarring. Additionally, in cases of a small, contracted (scleroatrophic) gallbladder, chronic inflammation and scarring can lead to shortening of the cystic plate (manifest as pucker sign). This reduces the distance between the gallbladder fundus (where dissection begins) and the right portal pedicle (where the cystic plate ends), increasing the risk of encountering the right portal pedicle early in the dissection. Such a scenario can result in extreme vasculobiliary injury. Therefore, during the FF approach, it is crucial for the surgeon to dissect as close to the gallbladder wall as possible, even if this risks entering the gallbladder lumen. Alternatively, the surgeon should promptly resort to SC as a bailout strategy [12, 15, 81].

#### Laparoscopic subtotal cholecystectomy

The CVS is one component of the culture of safety in cholecystectomy (COSIC), and a reliable bailout technique is another essential element of this culture. When the CVS cannot be achieved in difficult LC, a safe (avoiding BDI) and effective (eliminating the need for a second operation) bailout technique should be employed [82]. Among all bailout techniques, SC is the only option that fulfills both criteria, making it the preferred treatment in such scenarios [4].

Over the years, various techniques of SC have been described in the literature. In a systematic review published in 2013, Henneman et al. [83] identified four distinct laparoscopic SC techniques. The first two involve excising most of the anterior gallbladder wall while leaving the posterior wall in place, with the remaining gallbladder stump either closed or left open. The other two techniques involve partial resection of both the anterior and posterior gallbladder walls, with transection at Hartmann’s pouch, followed by either closure or leaving the pouch open.

To clarify whether SC leaves a remnant gallbladder, Strasberg et al. (2016) [82] introduced the terms reconstituting SC (where a closed remnant gallbladder is left behind) and fenestrating SC (where no such remnant gallbladder remains). In this respect, the ideal SC technique should meet several criteria: (1) it should not leave a gallbladder remnant that might be symptomatic and require future surgery; (2) it should have a low risk of biliary fistula, and any fistula that develops should resolve spontaneously within a short period; (3) it should be feasible laparoscopically; and (4) it should be performable by a general surgeon. However, these criteria often conflict with each other; fenestrating SC is less likely to leave a gallbladder remnant but carries a higher risk of biliary fistula, while the reverse is true for reconstituting SC.

In the systematic review of Henneman et al. (2013), the median operative time was 81.1 min, the conversion rate was 10.4%, and the median length of stay was 4.5 days. BDI occurred in 1.7% of patients. Postoperative bile leaks developed in 5.6% of patients with a closed cystic duct compared to 16% with an open cystic duct. Recurrent symptomatic stones occurred in four patients (2.2%), three of whom were managed with endoscopic papillotomy and one with reoperation. Postoperative ERCP was required for 2.7% of patients with a closed cystic duct compared to 16% with an open cystic duct. Percutaneous intervention was needed in 1.5% of cases (all with open cystic ducts) due to subhepatic collections. Reoperations were performed in 2.7% of cases (8 of 292 patients): three for intra-abdominal abscesses, two for persistent bile leaks, one for removal of an infected residual stone, and one for bleeding from the liver bed [83]. Long-term follow-up revealed gallstone recurrence in approximately 5% of patients, most of whom had undergone reconstituting SC. Since post-cholecystectomy syndrome (recurrent biliary pain) has been reported in 10–40% of patients after conventional LC, often due to recurrent or residual gallstones, then, the 5% recurrence rate of gallstones after SC is considered an acceptable outcome [84].

A more recent systematic review/meta-analysis of laparoscopic SC was published in 2015 by Elshaer et al. [85], including 1,231 patients. Of these, 73% underwent laparoscopic SC, while 17% had open SC. The cystic duct or gallbladder stump was closed in 91.4% and left open in 8.6% of patients. Bile leaks occurred in 42% of patients with an open cystic duct compared to 16.5% in those with a closed cystic duct, a statistically significant difference. Similarly, patients with an open cystic duct had higher incidences of subphrenic collections, retained stones (all requiring endoscopic or surgical intervention), reoperations for various indications, and 30-day mortality compared to those with a closed cystic duct. However, these differences were not statistically significant.

#### Conversion to open surgery

Although conversion to open surgery sacrifices the benefits of laparoscopy as a minimally invasive surgery, surgeons should not hesitate to convert when necessary. Indications for conversion include intolerance of pneumoperitoneum, severe inflammation hindering safe dissection, limited laparoscopic visibility, uncontrollable bleeding, and suspected or confirmed BDI [76]. Conversion allows for better exposure, control of bleeding, and placement of sutures when these cannot be achieved laparoscopically. However, conversion alone is not a bailout technique, as a difficult LC remains a difficult open cholecystectomy [86]. The bailout procedure is what follows conversion, with options including open cholecystectomy, open SC, or just open cholecystostomy tube insertion.

It is worth noting that over 30 years since the introduction of LC, the skills developed by young surgeons in performing open cholecystectomy are no longer attainable in current residency programs [4]. As a result, young surgeons may feel uncomfortable performing open cholecystectomy when LC cannot be safely completed. In such cases, seeking assistance from a more experienced colleague is strongly advisable, as these operations are often extremely challenging and associated with higher rates of BDI compared to routine LC [67]. A Dutch retrospective analysis found that open cholecystectomy resulted in more severe biliary injuries than LC [87]. Similarly, Kaplan et al. (2014) [88] and Davis et al. (2012) [89] reported BDI rates of 3.3% and 3.4% in open cholecystectomy groups respectively, whereas no BDIs occurred in laparoscopic SC groups.

Few studies have examined the economic implications of open conversion during emergency LC. Lengyel et al. [90] used the National Surgical Quality Improvement Program (NSQIP) and financial databases (2002–2009) to retrospectively compare long LC (LC with extended operative times) to converted LC. They found that hospital charges for long LC were 26% lower than for converted cases ($23,946 vs. $32,446; *P* < 0.01). Similarly, Shah et al. [91] retrospectively analyzed data of adult patients undergoing emergent LC from the National Inpatient Sample (NIS) (2007–2011) and found that the risk-adjusted hospital costs were 25.9% higher in converted cases, with an absolute difference of $23,358 (P < 0.05). Although operating room costs were higher in the LC group in both studies, overall costs were lower. Both studies emphasize that while open conversion is sometimes necessary and reflects sound surgical practice, prolonged operative time alone in difficult LC should not justify conversion as long as dissection is progressing safely [90, 91].

### Management of bile duct injury discovered during laparoscopic cholecystectomy

Unfortunately, BDIs are recognized intraoperatively in fewer than 25–30% of cases during LC [92]. This low recognition rate may be attributed to cognitive bias, as surgeons often rely on evidence that supports their initial perceptions while disregarding visual cues suggesting alternative diagnoses, as noted by Prasad et al. [93]. When a BDI is suspected during surgery, intraoperative cholangiography should be performed immediately to confirm the injury. If a BDI is confirmed, subsequent management depends on several factors, including: (1) the type and extent of the injury, (2) the presence of associated vascular injury, (3) the patient’s hemodynamic stability, (4) the operating surgeon’s skill and experience, and (5) the availability of a hepatopancreatobiliary surgical specialist. If hepatopancreatobiliary surgical expertise is not available, it is recommended to avoid further manipulation of the bile duct or conversion to open surgery. Instead, the surgeon should place a drain adjacent to the injured duct and transfer the patient to a specialized center after prior communication with the receiving team. This approach, often summarized as “drain now and fix later,” minimizes further damage and allows for definitive repair under optimal conditions [27].

Now, it’s well-established that the best outcomes of surgical repair of BDIs are achieved through early intervention by an experienced surgeon, with the first repair attempt typically yielding the most favorable results. A review of 88 patients with BDI following LC revealed a sharp contrast in success rates: 94% of repairs performed by specialist biliary surgeons were successful, compared to only 17% by non-specialists. Additionally, the length of stay was three times longer when managed by non-specialists (222 days versus 78 days). Morbidity and mortality rates were also significantly higher for non-specialists (58% and 1.6%, respectively) compared to specialists (4% and 0%) [94]. Similarly, Flum et al. (2003) reported poorer survival outcomes in patients whose repairs were performed by the injuring surgeon [95]. A recent single-center cohort study of 200 BDI patients further highlighted that on-table repair by a non- hepatopancreatobiliary surgeon was an independent risk factor for biliary stricture, recurrent cholangitis, revisional surgery, and overall morbidity [96].

On-table laparoscopic repair is recommended for minor leaks from the cystic duct or small (< 3 mm) ducts in the gallbladder bed, confirmed by intraoperative cholangiogram (Strasberg/Bismuth type A, C injuries). Similarly, laparoscopic repair of lateral injuries to the common bile duct or common hepatic duct without loss of continuity (type D) can be performed using fine monofilament absorbable sutures. Alternatively, abdominal drainage followed by endoscopic common bile duct stenting may be considered [97]. In cases of major BDI (type E), open conversion and construction of a tension-free Roux-en-Y hepaticojejunostomy is the standard approach. The bile duct stump should be healthy, free of inflammation, ischemia, and the anastomosis should be tension-free, constructed in a single layer using fine absorbable sutures. Notably, while the liver parenchyma derives approximately 75% of its oxygen supply from the portal vein, the biliary system relies essentially on hepatic arterial blood. Consequently, repairing BDI in the presence of associated arterial damage carries a significant risk of anastomotic failure or late stricture formation at the site of hepaticojejunostomy. If the right hepatic artery is also injured in a vasculobiliary injury, it is advisable to defer repair for several months to allow for the development of collateral circulation [95].

The acquisition of advanced laparoscopic skills in other areas of hepatopancreatobiliary surgery as in choledochal cyst resection and pancreaticoduodenectomy has encouraged surgeons to attempt total laparoscopic management of iatrogenic BDI. Over the past two decades, a few case reports have documented successful laparoscopic repair of BDIs. In 2005, Chowbey et al. reported the first successful laparoscopic repair of three out of four attempted BDIs. These patients presented 1 to 3 weeks post-LC and underwent Roux-en-Y hepaticojejunostomy, with a mean operative time of 268 min. One patient required an open revision of the anastomosis 18 months later due to recurrent cholangitis [98]. Despite the documented feasibility and safety of laparoscopic repair of BDIs in those few reports, the limited data reflect the significant challenges, technical complexity, and inherent limitations of the procedure. Consequently, most reported cases originated from centers with expertise in both hepatopancreatobiliary surgery and advanced laparoscopy.

Robotic-assisted surgery offers potential advantages over conventional laparoscopy, including 3D visualization with up to 10× magnification, elimination of surgeon tremors for enhanced stability, extreme ergonomics of wristed instruments, and ambidextrous handling. These features may help overcome some of the limitations associated with laparoscopic repair [99]. The largest series of minimally invasive BDI repairs to date was published by Cuendis-Velázquez et al. from Mexico in 2018. The study included 75 patients with type E1 to E5 BDIs, of whom 40 underwent laparoscopic repair and 35 underwent robotic-assisted repair. The overall morbidity rates were similar between the two groups, and the primary patency rate was 96% over a median follow-up of 28 months [100].

### Limitations of the study

This study has some limitations. As with all narrative reviews, this review may be subject to limitations in the completeness of the literature search and potential bias in interpretation. While the authors tried to include as much relevant literature as possible, it cannot be confirmed that all published studies were screened. Additionally, the interpretation of the included data may have been influenced by the authors’ perspectives, clinical experience, and knowledge. Finally, this review focused on difficult LC associated with acute and chronic cholecystitis. Several other scenarios of difficult LC, such as LC during pregnancy, LC in patients with morbid obesity, previous upper abdominal surgery, liver cirrhosis, and abnormally positioned gallbladders, were not addressed in this review.

## Conclusion

Difficult laparoscopic cholecystectomy is a common clinical scenario that can often be predicted before surgery. When encountered during surgery, difficult laparoscopic cholecystectomy requires a cautious and judicious approach by experienced surgeons in appropriate settings. In cases of acute cholecystitis with septic shock and in late-presenting acute cholecystitis, surgery should be avoided in favor of percutaneous transhepatic cholecystostomy, followed by delayed laparoscopic cholecystectomy several weeks later. In difficult laparoscopic cholecystectomy, the operating surgeon should seek a second opinion if he feels uncomfortable proceeding with surgery. Gallbladder drainage or aspiration is a safe bailout procedure for the impossible gallbladder. Intraoperative imaging, including intraoperative cholangiography, is essential for clarifying the unclear biliary anatomy, preventing, and identifying bile duct injuries. The fundus first (dome-down) approach is a valuable alternative to the standard (bottom-up) approach when the dissection of Calot’s triangle is risky or challenging. Currently, despite its drawbacks and potential complications, subtotal cholecystectomy is likely the best available bailout procedure. It can prevent bile duct injuries, and be completed laparoscopically without the need for a second operation in the majority of patients.

## Data Availability

No datasets were generated or analysed during the current study.
